# Osteogenic Differentiation and Proliferation of Apical Papilla Stem Cells Using Chitosan-Coated Nanohydroxyapatite and Bioactive Glass Nanoparticles

**DOI:** 10.1055/s-0043-1777044

**Published:** 2024-03-05

**Authors:** Sara Elshahat, Abeer Abdelhakim Elgendy, Tarek Elsewify

**Affiliations:** 1 Endodontic Department, Faculty of Dentistry, Ain Shams University, Cairo, Egypt; 2 Restorative Dental Sciences Department, College of Dentistry, Gulf Medical University, Ajman, UAE

**Keywords:** osteoinduction, osteoconduction, regenerative endodontic

## Abstract

**Objective** The aim of this study was to evaluate the osteogenic differentiation ability and proliferation of apical papilla stem cells (SCAPs) using chitosan-coated nanohydroxyapatite and bioactive glass nanoparticles.

**Materials and Methods** Hydroxyapatite, chitosan-coated nanohydroxyapatite, and bioactive glass 45S5 nanoparticles were prepared and characterized using a transmission electron microscope and X-ray diffraction. SCAPs were harvested from freshly extracted impacted wisdom teeth, cultured, and characterized using flow cytometric analysis. Tested nanomaterials were mixed and samples were classified into five equal groups as follows: negative control group: SCAP with Dulbecco's modified eagle's medium, positive control group: SCAP with inductive media, first experimental group: nanohydroxyapatite with SCAP, second experimental group: chitosan-coated nanohydroxyapatite with SCAP, third experimental group: bioactive glass nanoparticles with SCAP. Osteoblastic differentiation was assessed using an alkaline phosphatase (ALP) assay. Receptor activator of nuclear factor kappa beta ligand (RANKL) expression was evaluated using specific polyclonal antibodies by fluorescence microscope. The proliferation of SCAP was assessed using cell count and viability of trypan blue in addition to an 3-(4,5-dimethylthiazol-2-yl)-2,5-diphenyltetrazolium bromide (MTT) assay.

**Results** Isolated SCAP showed a nonhematopoietic origin. Chitosan-coated nanohydroxyapatite showed the highest ALP concentration followed by nanobioactive glass, nanohydroxyapatite, and negative control. Chitosan-coated nanohydroxyapatite showed the highest H score followed by nanobioactive glass, nanohydroxyapatite, and negative control in RANKL expression. Chitosan-coated nanohydroxyapatite showed the highest viable cell count.

**Conclusion** SCAP isolation is achievable from extracted fully impacted immature third molars. All tested biomaterials have the ability to induce osteogenic differentiation and proliferation of SCAP. Composite nanoparticle materials show better osteogenic differentiation and proliferation of SCAP than single nanoparticles.

## Introduction

Regenerative endodontic procedures are considered nowadays the ideal treatment for necrotic immature permanent teeth. These procedures will allow for hard tissue formation completing the root structure in length and thickness. Stem cells, growth factors, and scaffolds are the three major components of regenerative endodontic procedures. 1

Dental pulp stem cells, stem cells from human exfoliated deciduous teeth, periodontal ligament stem cells, and stem cells of the apical papilla can now be isolated. Apical papilla stem cells (SCAPs) are located at the apical part of immature teeth. 2 Osteogenic differentiation of SCAPs and the formation of osteoblast and osteoblast-like cells have been demonstrated in addition to the formation of new hard tissue. 3

Bioactive glass (BG) has been successfully used with implant placement and to treat pathological periodontal bony defects owing to its superior biocompatibility, osteo-conductive and -inductive properties. BG has the ability to modify osteoblastic gene expression in a way that properly controls cell proliferation and differentiation. 4 5 Applying the BG in a nano-sized particles, 45S5 BG, has increased its osteoconduction, osteoinduction properties and allowed for its use in bone tissue engineering. 6 7

Hydroxyapatite shows excellent biocompatibility. Preparing it in the nanoscale yielded superior biologic properties when used as a scaffold owing to particle size similar to that of the natural hydroxyapatite. Nanohydroxyapatite has yielded promising results in various regenerative procedures and led to the creation of various composite scaffolds. 8

Chitosan, extracted from crustaceans, has been recently used and tested in various endodontic applications owing to its excellent biologic behavior. It is a cationic polymer that demonstrates good antimicrobial properties. 9 10 11 Composite scaffolds such as chitosan/hydroxyapatite have shown promising osteconductivity, gaining the advantage of both materials used. 12

The effect of hydroxyapatite coated by chitosan nanoparticles and BG nanoparticles on the osteogenic differentiation and proliferation of stem cells of the apical papilla has not yet been evaluated. Therefore, the aim of this study was to evaluate the effect of hydroxyapatite coated by chitosan nanoparticles and BG nanoparticles on the osteogenic differentiation and proliferation ability of stem cells of the apical papilla. The null hypothesis tested is that there is no significant difference between the hydroxyapatite coated by chitosan nanoparticles and BG nanoparticles on the osteogenic differentiation and proliferation of stem cells of the apical papilla.

## Materials and Methods

### Preparation of Nanomaterials

#### Bioactive Glass 45S5 Nanoparticles

The sol-gel method was adopted to prepare BG 45S5 nanoparticles from a colloidal solution of 45S5 composition (45 mol% SiO _2_ , 24.5 mol% CaO, 24.5 mol% Na _2_ O and 6 mol% P _2_ O _5_ ). 13 Ceramic powder was produced from the gel after heating.

#### Hydroxyapatite Nanoparticles

Ammonium hydroxide and calcium nitrate were used to synthesize the hydroxyapatite nanoparticles following the methodology previously described by Cengiz et al. 14

#### Chitosan-Coated Nanohydroxyapatite

Preparation of the composite chitosan-coated nanohydroxyapatite was done following Nikpour et al methodology. 15 Powder was obtained after freeze-drying of the mixture.

**Fig. 1:**
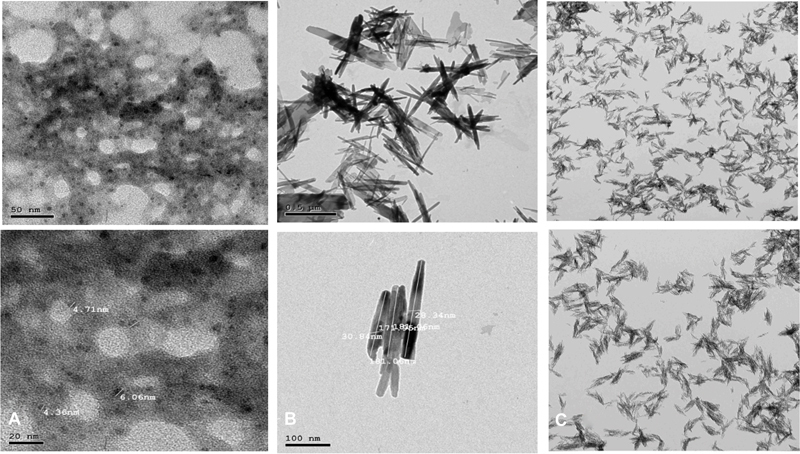
Transmission electron microscopic image of ( **A** ) bioactive glass 45S5 nanoparticles, ( **B** ) hydroxyapatite nanoparticles, and **(C)** chitosan-coated nanohydroxyapatite nanoparticles.

Characterization of all of the prepared nanoparticles was done using high-resolution transmission electron microscopy (TEM) and X-ray Powder Diffraction (XRD) with 2 thetas (10 ^ᴼ^ -70 ^ᴼ^ ), with a scanning speed of 1°/min and minimum step size 2Theta: 0.001 at wavelength (Kα) = 1.54614ᴼ 16 as shown in Fig. 1 .

### Stem Cells Harvesting and Culture

SCAPs were harvested and cultured from freshly extracted wisdom teeth of three patients after obtaining an informed consent. Inverted phase contrast microscope was used to check for growth and/or contamination. 17

#### SCAP Characterization

Flow cytometric analysis was performed using the protocol published earlier on Navios software. 17

#### SCAP Culture

Cells cultured in complete culture media and harvested after the third passage. The harvested SCAPs were cryopreserved at °80°C for further analysis. 17

All tested nanomaterials were mixed according to manufacturer instructions. The samples were classified to five equal groups:

- **Negative control group:** SCAP with Dulbecco's Modified Eagle Medium (DMEM).
- **Positive control group:** SCAP with inductive media (OM).
- **Group I:** Nanohydroxyapatite (NHAP 10 µg/mL) with SCAP.
- **Group II:** Chitosan-coated nanohydroxyapatite (NHAP/chitosan 10 µg/mL) with SCAP.
- **Group III:** BG nanoparticles (NBG 500 µg/mL) with SCAP.

For osteoblastic differentiation, six-well plates were used to culture stem cells of the apical papilla OM seeded at 4.5 × 10 ^5^ cells/well. Plates were incubated for a period of 72 hours at 37°C and 5% CO _2_ . The activity of alkaline phosphatase (ALP) was measured using enzymatic dephosphorylation by ALP assay kit. For testing the expression of RANKL for SCAP, the cells were examined using specific polyclonal antibody by fluorescence microscope.

Regarding evaluation of the proliferation, the SCAPs were stained by trypan blue and counted by hemocytometer to estimate the number of dead cells. The MTT assay was performed using the Vybrant MTT Cell Proliferation Assay Kit. Cell viability was determined by measuring the optical density at 570 nm on a spectrophotometer.

### Statistical Analysis

Mean and standard deviation values of each group were calculated. Shapiro–Wilk test and Levene's test were used to test for normality of the data. One-way analysis of variance test was run followed by Tukey's post hoc test as the data was normally distributed. The significance level was set at *p* -value less than0.05. Statistical analysis was performed with Statistical package for Social Science software.

## Results

**Fig. 2:**
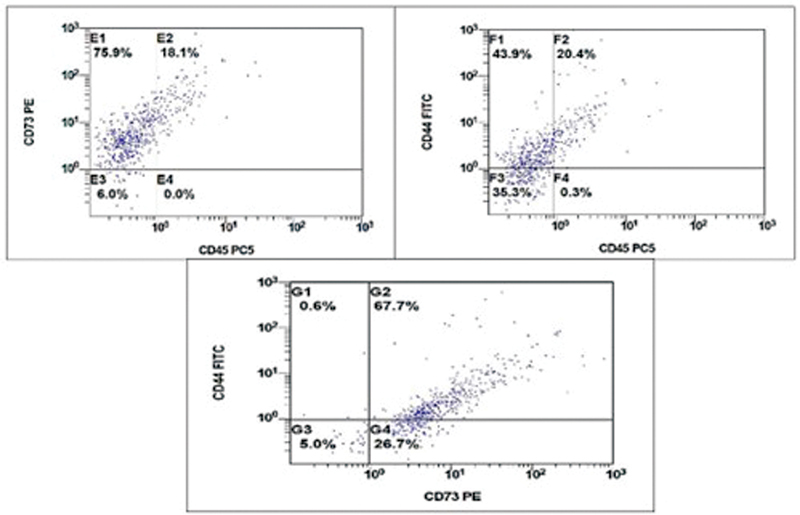
Flow cytometry (FCM) dot plots showing the gate protocol for apical papilla stem cells (SCAPs). The SCAPs were stained with stem cell markers (CD73, CD44, and CD45). The CD73 and CD44 positive cells were gated in corresponding to CD45.

The observed results of the characterized SCAP revealed that the cells showed double bright surface expression of CD44/CD73 and failed to express CD45, indicating a nonhematopoietic origin as shown in Fig. 2 .

**Fig. 3:**
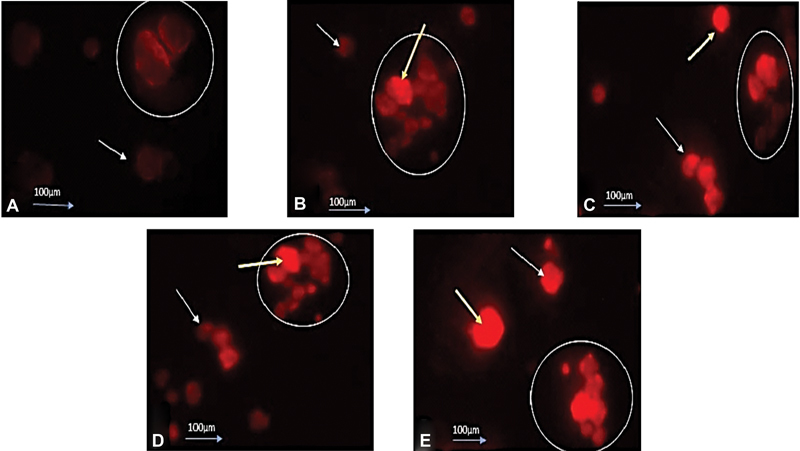
Photomicrograph showing expression of RANKL protein in differentiated apical papilla stem cells (SCAPs), the photos were captured by LABOMED Immunofluorescence microscopes. ( **A** ) Negative control cells shows small colonies of cells that showed a homogenous faint expression of RANKL, the expression was localized to the cell membrane. ( **B** ) Positive control cells with increased number of osteoblasts like colonies which are presented with dense homogenous expression of RANKL. However, the SCAP cultured with nanohydroxyapatite (NHAP) ( **C** ), NHAP/chitosan ( **D** ) and bioactive glass nanoparticles ( **E** ), showed a merged large colony of osteoblast like cells with dense homogenous membranous and nuclear expression of RANKL. The magnification power is 10x.The white circles highlight the osteoblast like colonies, white arrow: membranous expression of RANKL, yellow arrow: dense nuclear expression of RANKL.

NHAP/chitosan showed the highest ALP concentration followed by NBG, NHAP, and DMEM-NC as shown in Table 1 . RANKL expression results are shown in Table 2 and Fig. 3 where NHAP/chitosan showed the highest H score followed by NBG, NHAP, and DMEM-NC. NHAP/chitosan showed the highest viable cell count as shown in Table 3 . NHAP/chitosan showed the highest viable count also using the MTT assay, although the difference was not statistically significant as shown in Table 4 .

**Table 1:** Mean ± SD and *p* -values of ALP concentration of all tested groups

	(OM-PC)	(NHAP 10 µg/mL)	NHAP/chitosan (10 µg/mL]	NBG (500 µg/mL)	(DMEM-NC)
Mean ± SD	77.86 ± 0.15 ^b^	68.72 ± 0.13 ^c^	82.90 ± 0.10 ^a^	70.36 ± 0.10 ^d^	55.18 ± 0.8 ^e^
*p* -Value	<0.001				

Abbreviations: ALP, alkaline phosphatase; NBG, bioactive glass nanoparticles; NHAP, nanohydroxyapatite; SD, standard deviation.

Means with different letters were statically significant.

**Table 2:** Mean ± SD and *p* -values of IF assay of all tested groups

	(OM-PC)	(NHAP 10 µg/mL)	NHAP/chitosan (10 µg/mL)	NBG (500 µg/mL)	(DMEM-NC)
Mean ± SD	82.67 ± 1.53 ^c^	67.67 ± 2.52 ^d^	180.67 ± 4.04 ^a^	154.67 ± 4.16 ^b^	17.68 ± 1.52 ^e^
*p* -Value	<0.001				

Abbreviations: IF, immunofluorescence; NBG, bioactive glass nanoparticles; NHAP, nanohydroxyapatite; SD, standard deviation.

Means with different letters were statically significant.

**Table 3:** Total, dead, viable cell counts, and mean ± SD values for tested groups

	Total cell count	Dead cell count	Viable cell count	% Viability
(OM-PC)	60.7 × 10 ^5^ ‎ ± 34.2 × 10 ^5 b^	7.34 × 10 ^3^ ± 1.89 × 10 ^3b^	60.6 × 10 ^5^ ± 34.2 × 10 ^5 b^	99.879
NHAP (10 µg/mL)	157 × 10 ^5^ ± 33.8 × 10 ^5 a^	5.28 × 10 ^3^ ± 0.88 × 10 ^3 b^	157 × 10 ^5^ ± 33.8 × 10 ^5 a^	99.966
NHAP/Ch (10 µg/mL)	218 × 10 ^5^ ± 7.21 × 10 ^5 b^	1.64 × 10 ^3^ ± 0.12 × 10 ^3 b^	218 × 10 ^5^ ± 7.21 × 10 ^5 a^	99.996
NBG (500 µg/mL]	198.7 × 10 ^5^ ± 69.8 × 10 ^5 a^	18.9 × 10 ^3^ ± 7.80 × 10 ^3 a^	198.7 × 10 ^5^ ± 69.8 × 10 ^5 a^	99.905
(DMEM-NC)	4.647 × 10 ^5^ ± 1.147 × 10 ^5 b^	4.11 × 10 ^3^ ± 4.41 × 10 ^3 a^	4.6 × 10 ^5^ ± 1.1 × 10 ^5 b^	99.116
*p* -Value	<0.001	0.004	<0.001	

Abbreviations: Ch, chitosan; NBG, bioactive glass nanoparticles; NHAP, nanohydroxyapatite; SD, standard deviation.

Means with different letters were statically significant.

**Table 4:** Mean ± SD and *p* -values of viability test (MTT assay)

	(OM-PC)	(NHAP 10 µg/mL)	NHAP/chitosan (10 µg/mL)	NBG (500 µg/mL)	(DMEM-NC)
Mean ± SD	0.930 ± 0.042 ^b^	1.502 ± 0.243 ^a^	1.645 ± 0.23 ^a^	1.551 ± 0.292 ^a^	0.821 ± 0.019 ^b^
*p* -Value	<0.001				

Abbreviations: NBG, bioactive glass nanoparticles; NHAP, nanohydroxyapatite; SD, standard deviation.

Means with different letters were statically significant.

## Discussion

Proper management and long-term success of nonvital immature permanent teeth continue to be a challenge for clinicians. The ability to regenerate a pulp or pulp-like structure that can lay down hard tissue structure increasing the root length and thickness will increase the tooth's fracture resistance and maintain it in function for a longer duration. 18

During various regenerative endodontic procedures, mesenchymal stem cells have been demonstrated related to immature teeth in addition to mature ones. The origin of such mesenchymal stem cells is believed to be the apical papilla, bone, Periodontal ligament (PDL), and/or granulomas. 19

Apical papilla can be defined as the loosely attached soft tissue related to the root end of immature teeth. This apical papilla is separated from the dental pulpal tissue by a cell rich zone. The dental pulpal tissue shows more cellular and vascular elements than does the apical papilla. 2

SCAPs were first isolated by Sonoyama et al. SCAPs are derived from an embryonic neural crest-like tissue, located at the root end of immature teeth. In contrast to other isolated types of stem cells, SCAPs demonstrate impressive odontogenic differentiation and proliferation in addition to massive dentinogensis. 2 20

Under favorable conditions, mineral trioxide aggregates have the ability to stimulate the proliferation and differentiation of SCAPs resulting in hard tissue formation. However, the effect of other materials on SCAPs is not well studied. 21 BG, hydroxyapatite, and chitosan have shown promising results when tested for their biologic effect on dental pulp stem cells and mesenchymal stem cells. 22-27 The aim of the current study was to investigate the effect of nanohydroxyapatite coated by chitosan and nano-BG on osteogenic differentiation and proliferation of stem cells of the apical papilla.

Trypan blue was used for counting the viable cells in the current study owing to its characteristic ability to stain only the dead cells following penetration of its cell membrane. 28 29 The MTT assay was used due to its capability to determine the mitochondrial activity. 30 31 ALP enzyme activity assays were used as a measure of SCAP differentiation into osteoblast-like cell as it is considered as a characteristic marker for bone-forming cell differentiation. 32 Immunofluorescence assay is considered as one of the most reliable tests that helps elaborate specific protein of interest through antigen-antibody reaction. 33 The RANK-L concentration is proportional to the number of osteogenic cells because it is deemed mandatory for its differentiation. 34 35

Our results of SCAP characterization come in full agreement with Kang et al who also confirmed the nonhemopoietic origin of the stem cells by lack of CD45 expression. 36

The superior results of NHAP/chitosan group regarding the ALP and RANKL come in full agreement with Kong et al. 37 This superiority could be attributed to the composite nature of this group that allowed for better mineralization and differentiation. This can be explained by the increased levels of calcium phosphate and calcium carbonate. 38 This also comes in agreement with Ge et al 39 who tested this composite on periodontal ligament stem cells differentiation. They attributed their results to increased concentration of the calcium and phosphate ions.

The NHAP/chitosan group also showed superior results in osteogenic differentiation and proliferation potential of SCAP. This might be because of the chitosan coating that directly stimulates progenitor cell differentiation at the mRNA level of ALP enzyme. 40 41 42 This superiority of the composite group comes in full agreement with Kong et al 37 who attributed this to the topography and quantity. Also, this finding is similar to that obtained by Ge et al 39 on periodontal ligament stem cells explained on basis of surface chemistry and geometry. Similar results were also obtained by Tondnevis et al 43 on dental pulp stem cells.

BG showed significant effect on SCAP viability and osteogenic differentiation compared to the negative control group. This is consistent with Wang et al 44 who tested BG on bone marrow stem cells. This could be simply explained by the increased ion release, specifically calcium ions that attracts different cells.

The nanohydroxyapatite group also showed significant effect on SCAP viability and osteogenic differentiation in compared to the negative control group. This finding is in agreement with Yang et al 25 who tested it on mesenchymal stem cells. This could be explained on the basis of the nanoparticle size that greatly affects its behavior in addition to the increased calcium ions release that increases cell mineralization.

Nanohydroxyapatite coated by chitosan, nanobioactive glass and nanohydroxyapatite as biomaterials, proved to enhance the osteogenic differentiation and proliferation of SCAP. This could improve the regenerative procedure in endodontic as osteogenic differentiation enhances lesion healing and laying down hard tissue structure that might be dentin like.

## Conclusion

Within the limitations of this *in vitro* study, it can be concluded that isolation of SCAP can be done from extracted fully impacted immature third molars. All tested biomaterials have the ability to induce osteogenic differentiation and proliferation of SCAP. Chitosan-coated nanohydroxyapatite biomaterial has increased ability for differentiation of SCAP to osteoblasts. Chitosan-coated nanohydroxyapatite biomaterial has increased ability for proliferation of SCAP proved by upregulated cell viability.
